# 6-Pentyl-α-Pyrone from *Trichoderma gamsii* Exert Antioxidant and Anti-Inflammatory Properties in Lipopolysaccharide-Stimulated Mouse Macrophages

**DOI:** 10.3390/antiox12122028

**Published:** 2023-11-22

**Authors:** Jae Sung Lim, Joo-Hyun Hong, Da Young Lee, Xiangying Li, Da Eun Lee, Jeong Uk Choi, Kwang Youl Lee, Ki Hyun Kim, Young-Chang Cho

**Affiliations:** 1 College of Pharmacy and Research Institute of Pharmaceutical Sciences, Chonnam National University, Gwangju 61186, Republic of Korea; dr.jslim7542@gmail.com (J.S.L.); dlekdud0914@naver.com (D.Y.L.); lxyoung825@naver.com (X.L.); 2 School of Pharmacy, Sungkyunkwan University, Suwon 16419, Republic of Korea; jhong526@ildong.com (J.-H.H.); allag8201@naver.com (D.E.L.); 3 Research Laboratories, ILDONG Pharmaceutical Co. Ltd., Hwaseong 18449, Republic of Korea; 4 College of Pharmacy, Kyung Hee University, Seoul 02447, Republic of Korea; cju0667@khu.ac.kr

**Keywords:** 6-pentyl-α-pyrone, anti-inflammatory, antioxidant, MAPK, NF-κB, Nrf2

## Abstract

Filamentous fungi produce several beneficial secondary metabolites, including bioactive compounds, food additives, and biofuels. *Trichoderma*, which is a teleomorphic *Hypocrea* that falls under the taxonomic groups Ascomycota and Dikarya, is an extensively studied fungal genus. In an ongoing study that seeks to discover bioactive natural products, we investigated potential bioactive metabolites from the methanolic extract of cultured *Trichoderma gamsii.* Using liquid chromatography–mass spectrometry (LC–MS), one major compound was isolated and structurally identified as 6-pentyl-α-pyrone (6PP) based on nuclear magnetic resonance data and LC–MS analysis. To determine its antioxidant and anti-inflammatory activity, as well as the underlying mechanisms, we treated lipopolysaccharide (LPS)-stimulated Raw264.7 mouse macrophages with 6PP. We found that 6PP suppresses LPS-induced increase in the levels of nitric oxide, a mediator of oxidative stress and inflammation, and restores LPS-mediated depletion of total glutathione by stabilizing nuclear factor erythroid 2-related factor 2 (Nrf2), an antioxidative factor, and elevating heme oxygenase-1 levels. Furthermore, 6PP inhibited LPS-induced production of proinflammatory cytokines, which are, at least in part, regulated by heme oxygenase-1 (HO-1). 6PP suppressed proinflammatory responses by inhibiting the nuclear localization of nuclear factor kappa B (NF-κB), as well as by dephosphorylating the mitogen-activated protein kinases (MAPKs). These results indicate that 6PP can protect macrophages against oxidative stress and LPS-induced excessive inflammatory responses by activating the Nrf2/HO-1 pathway while inhibiting the proinflammatory, NF-κB, and MAPK pathways.

## 1. Introduction

Host inflammatory responses that protect from invading pathogens, including bacterial infections, are mainly mediated by innate immune cells, such as neutrophils, macrophages, and dendritic cells [1,2]. Of these, macrophages, which detect invading pathogens via specific receptors, especially toll-like receptor 4, which recognizes lipopolysaccharides (LPS), a constituent of Gram-negative bacterial cell walls, are key inflammation mediators [3]. LPS-interacting macrophages trigger inflammation by expressing proinflammatory mediators, such as tumor necrosis factor (TNF)-α, interleukin (IL)-1β, IL-6, and nitric oxide (NO) [4,5]. Oxidative stress is also closely associated with the development of chronic inflammatory diseases [6]. Moreover, oxidative stress and inflammatory responses are interdependent because reactive oxygen species induce the release of proinflammatory factors, which, in turn, trigger oxidative stress [7]. To maintain homeostasis in macrophages, the stabilization of nuclear factor erythroid 2-related factor 2 (Nrf2) against oxidative stress is crucial. In response to oxidative stress and LPS, Nrf2 translocates to the nucleus and drives the expression of antioxidant genes, such as heme oxygenase-1 (HO-1) and NAD(P)H dehydrogenase [quinone] 1 [8]. Under excessive oxidative stress and proinflammatory stimuli, the homeostasis of Nrf2 and other anti-inflammatory factors is disrupted, resulting in oxidative stress- and inflammation-mediated diseases, such as asthma, rheumatoid arthritis, hepatitis, and ulcers [9,10,11]. Therefore, the control of macrophage activity is a potential strategy for the development of effective inflammatory disease treatments.

*Trichoderma* species, which are teleomorphic *Hypocrea*, fall under the taxonomic groups Ascomycota and Dikarya and are rich in secondary metabolites, including >1000 known compounds with a wide range of structures [12]. Some *Trichoderma* species, such as *Trichoderma reesei*, *Trichoderma harzianum*, *Trichoderma atroviride*, *Trichoderma virens*, *Trichoderma asperellum*, and *Trichoderma asperelloides* have a high industrial value and have been directly used as biological control agents [13]. *Trichoderma* species also produce various enzymes with potential biocontrol activity, including cell wall degradation, biotic and abiotic stress tolerance, hyphal growth, and anti-plant pathogen effects. Consequently, cell wall degrading enzymes from *Trichoderma* species can be used for the development of commercial products [14]. *Trichoderma gamsii (T. gamsii*) strongly inhibits the growth of wood-damaging fungi by releasing volatile metabolites and is known as a key producer of biological control agents [15]. Previous studies of the secondary metabolites from *T. gamsii* identified several cytochalasans with distinct structures, such as trichalasins C and D, aspochalasins D, M, and P, and trichoderones A and B [16,17]. Several of these compounds exhibit notable cytotoxicity [16]. Moreover, *T. gamsii* possesses intriguing metabolites, such as trichoderpyrone (a unique polyketide hybrid with a cyclopentenone-pyrone framework [18]), trichoderamides A and B (a pair of unique stereoisomers), and two novel compounds, trichodenols A and B [19]. Thus, investigation of the secondary metabolites from *T. gamsii* holds great promise.

In an ongoing study to identify new bioactive compounds from various natural materials, including microbes [20,21,22,23,24], we investigated potential bioactive metabolites from the methanolic extract of cultured *T. gamsii*. Using column chromatography and high-performance liquid chromatography (HPLC), coupled with liquid chromatography–mass spectrometry (LC–MS), we isolated one major compound, which was identified as 6-pentyl-α-pyrone (6PP).

To determine its antioxidant and anti-inflammatory effects, we treated Raw264.7 macrophages with 6PP at various concentrations in the absence or presence of LPS and then examined its antioxidant and anti-inflammatory effects, as well as the underlying mechanisms. Our findings indicate that 6PP exerts antioxidant and anti-inflammatory effects by activating the Nrf2/HO-1 and the nuclear factor kappa B (NF-κB) signaling pathways while inhibiting the mitogen-activated protein kinase (MAPK) signaling pathway.

## 2. Materials and Methods

### 2.1. General Experimental Procedures

Nuclear magnetic resonance (NMR) spectra were acquired using a Bruker Avance III HD 500 NMR spectrometer at 500 MHz (^1^H), and chemical shifts were determined in ppm (δ). Preparative HPLC was done using a Waters 1525 Binary HPLC pump equipped with a Waters 996 photodiode array detector (Waters Corporation, Milford, CT, USA). LC–MS analysis was done on an Agilent 1200 Series HPLC system (Agilent Technologies, Santa Clara, CA, USA) equipped with a diode array detector and a 6130 Series ESI mass spectrometer on an analytical Kinetex C18 Å column (100 mm × 2.1 mm, 5 μm, Phenomenex) at a flow rate of 0.3 mL/min. Spots were detected on a thin-layer chromatography (TLC) plate under UV light or upon heating after spraying with anisaldehyde–sulfuric acid. Column chromatography was done using a silica gel 60 (230–400 mesh, Merck, Darmstadt, Germany). Merck precoated silica gel F254 plates and RP-18 F254s plates were used for TLC.

### 2.2. Fungal Material

*T. gamsii* KUC1747 was purchased from the Korea University Culture Collection (KUC).

### 2.3. T. gamsii Culture and Isolation of 6PP

*T. gamsii* was pre-cultured on potato dextrose agar medium ([PDA], BD Difco, Tucker, GA, USA) at 25 °C for five days in the dark. Three inoculums were transferred from the margin of a PDA plate to 5 L PDA plates and incubated at 25 °C for seven days, followed by extraction using 10 L of MeOH to obtain the MeOH extract. The crude methanol (MeOH) extract (3.5 g) was successively partitioned using ethyl acetate (EtOAc), and the resulting EtOAc fraction (1.2 g) was subjected to fractionation using silica gel column chromatography (diameter: 2 × 40 cm) on hexane/EtOAc gradients of 1:0, 1:100, 1:10, 1:1 and 0:1. This generated five EtOAc sub-fractions based on the TLC profiles obtained. The sub-fractions were eluted using hexane/ethyl acetate (1:10) and further separated via chromatography using Sephadex LH-20 (diameter: 2 × 35 cm, CHCl_3_: MeOH = 1:1) on a C18 column (diameter: 1 × 26 cm) using a 20–100% MeOH gradient and preparative HPLC (YMC J’sphere ODS-H80, 4 μm, 250 × 20 mm i.d., at a flow rate of 4.0 mL/min in 50–70% MeOH, for 50 min), which isolated one major compound (50 mg).

### 2.4. DPPH (2,2’-Diphenyl-1-picrylhydrazyl radical) Assay

6PP was diluted in 100% MeOH to indicated concentrations, respectively, after which 100 µL of each diluent was transferred to 96-well plates. Afterward, 100 µL of 0.2 mM DPPH solution was added and incubated for 30 min in a 37 °C incubator. After incubation, absorbance was measured at 517 nm using a microplate reader (Synergy HTX, BioTek Instruments Inc., Winooski, VT, USA). The radical scavenging activity was then calculated as (Abssample − AbsMeOH)/AbsMeOH and expressed as relative values. Quercetin (Sigma-Aldrich, St. Louis, MO, USA) was used as the positive control.

### 2.5. Cell Culture

Raw264.7 cells (ATCC, #TIB-71, Fairfield, NJ, USA) were cultured in high glucose Dulbecco’s Modified Eagle’s Medium (DMEM) (#LM0001-05, WELGENE Inc., Gyeongsan, Republic of Korea) supplemented with 10% fetal bovine serum (#16000044, Gibco, Waltham, MA, USA), 100 IU/mL of penicillin, and 100 µg/mL of streptomycin (#30-002-cl, Corning Inc., Corning, NY, USA). The cells (2nd to 3rd passage) were cultured at 37 °C in a humidified CO_2_ incubator.

### 2.6. Cell Viability Assay

Raw264.7 cells were seeded in 96-well plates at a density of 2 × 10^4^ cells/well and then cultured in the presence of 6PP at 12.5, 25, 50, 100, and 200 µM for 24 h. Cell viability assays were then performed using an EZ-Cytox cell viability assay kit (#EZ-1000, DoGenBio, Seoul, Republic of Korea). Briefly, the cells were incubated with the EZ-Cytox solution, which contained water-soluble tetrazolium salt, for 30 min, followed by absorbance reading at 450 nm on a microplate reader (Synergy HTX, BioTek Instruments Inc.). Cell viability was calculated as a percentage relative to the untreated group (100%).

### 2.7. Glutathione (GSH) Assay

GSH levels were assessed using a glutathione assay kit (#703002, Cayman Chemical Company, Ann Arbor, MI, USA) according to manufacturer instructions. Raw264.7 cells were seeded in 12-well plates at a density of 2 × 10^5^ cells/well and then pretreated with 6PP at 12.5, 25, 50, 100, and 200 µM for 2 h. They were then stimulated with LPS (0.5 µg/mL) for 24 h, washed with cold phosphate-buffered saline (PBS), and then sonicated in cold 2-(*N*-morpholino) ethane sulfonic acid (MES) buffer (50 mM), which was provided in the GSH assay kit. The samples were then centrifuged at 10,000× *g* for 15 min at 4 °C. The supernatant was then mixed with an equal volume of 10% meta-phosphoric acid (#239275, Sigma–Aldrich). The solution was then reacted with an enzyme cocktail containing nicotinamide adenine dinucleotide phosphate (NADPH), glutathione reductase, and 5,5′-dithio-bis-2-nitrobenzoic acid in a 96-well plate to quantify total GSH and glutathione disulfide (GSSG). For GSSG measurement, the supernatant was incubated with 1M 2-vinyl pyridine (#13229-2, Sigma-Aldrich) at room temperature (RT) for 1 h before the enzymatic reaction. Following 25 min incubation in the dark, absorbance was read at 410 nm on a microplate reader (Synergy HTX, BioTek Instruments).

The levels of GSH and GSSG were calculated using the formula:

Total GSH or GSSG (μM) = [(absorbance of the sample) − (y-intercept)]/(slope) × 2 × sample dilution.
GSH (μM) = (Total GSH) − 2 × (GSSG)

### 2.8. Measurement of Nitric Oxide (NO) Levels

Raw264.7 cells were seeded in 12-well plates at a density of 2.5 × 10^5^ cells/well and pretreated with 6PP at 12.5, 25, 50, 100, and 200 µM for 2 h. They were then stimulated with LPS (0.5 µg/mL) for 24 h. Next, 100 µL of the cell culture supernatant was mixed with an equal volume of Griess reagent containing 1% sulfanilamide, 0.1% N-1-naphthyl ethylenediamine dihydrochloride, and 2.5% phosphoric acid, and then incubated in the dark for 10 min. Absorbance was then measured at 540 nm using a microplate reader (Synergy HTX, BioTek Instruments).

### 2.9. Western Blot Analysis

Western blot analysis was done as previously described [25]. Briefly, the cells were pretreated with 6PP at 25, 50, 100, and 200 µM for 2 h and then stimulated with LPS (0.5 µg/mL) for 15 min or 24 h. Total proteins were extracted using a rapid immunoprecipitation assay buffer (#RC2002-050-00, Biosesang, Seongnam, Republic of Korea) supplemented with a protease inhibitor cocktail and phosphatase inhibitor cocktails II and III (#P8340, #P5726, and #P0044, respectively, Sigma–Aldrich) on ice. After protein quantification, total proteins were denatured, resolved using SDS–PAGE, and transferred onto nitrocellulose membranes. The membranes were blocked using 5% nonfat dry milk (#SKI500, LPS Solution, Daejeon, Republic of Korea) in Tris-buffered saline (25 mM Tris-HCl pH 8.0 and 125 mM NaCl) containing 0.1% Tween-20 (TBS-T) at RT, for 1 h. The membranes were then incubated with the indicated primary antibodies at 4 °C overnight. They were then washed thrice using TBS-T and incubated with horseradish peroxidase-conjugated anti-mouse or anti-rabbit secondary antibodies at RT for 1 h. The protein signal was then developed using enhanced chemiluminescence (#BWP0200, Biomax, Seoul, Republic of Korea) and imaged on a chemiluminescence imaging system (Amersham Imager 680, GE Healthcare, Chicago, IL, USA). Protein band densitometric analysis was determined using Amersham software (ImageQuant TL ver 8.2) (GE Healthcare, Chicago, IL, USA). The following antibodies were used for western blot analyses: anti-iNOS (#610332, BD Biosciences, San Diego, CA, USA), anti-HO-1 (#sc-390991), anti-β-actin (#sc-47778), anti-p38 (#sc-7972), and anti-p44/42 (#sc-514302; Santa Cruz Biotechnology Inc., Dallas, TX, USA), and anti-Nrf2 (#12721), anti-SAPK/JNK (#9252), anti-phospho-SAPK/JNK (#9251), anti-phospho-p38 (#9211), anti-phospho-p44/42 (#9101), anti-p-IκB (#9246), anti-IκB (#9242), anti-mouse (#7076), and anti-rabbit (#7074; Cell Signaling Technology, Danvers, MA, USA).

### 2.10. Enzyme-Linked Immunosorbent Assay (ELISA)

Raw264.7 cells were seeded in 12-well plates at a density of 2.5 × 10^5^ cells/well, pretreated with 6PP at 25, 50, 100, and 200 µM for 2 h, and then stimulated with LPS (0.5 µg/mL) for 24 h. Cytokine levels in the culture media were then measured using an ELISA kit according to the manufacturer’s instructions. Briefly, purified anti-IL-1β (#14-7012-85) and anti-TNF-α (#14-7423-85; Thermo Fisher Scientific Inc., Waltham, MA, USA) and anti-IL-6 (#554400, BD Pharmingen, San Diego, CA, USA), antibodies were used to coat 96-well plates at 4 °C, overnight. The plates were then washed thrice using PBS-T (0.05% Tween-20 in PBS) and blocked for 1 h at RT, using 1% bovine serum albumin (BSA) in PBS. Following incubation of the supernatants for 2 h at RT, the plates were washed thrice using PBS-T. Next, detection antibodies were added, followed by incubation for 1 h at RT. The plates were then washed four times with PBS-T, incubated with streptavidin-conjugated alkaline phosphatase (AKP, #554065, BD Pharmingen) for 30 min at RT, and then washed four times with PBS-T. Finally, the plates were incubated in the dark with a substrate buffer (pH 9.8) containing 10% diethanolamine (#3032-4400), 0.1% MgCl_2_·6H_2_O (#5503-44), and 0.2% NaN_3_ (#7530-4105; Daejung Chemicals & Metals Co., Siheung, Republic of Korea), and 4-nitrophenyl phosphate (#N2765, Sigma-Aldrich). The reaction was terminated using 1 N NaOH, followed by an absorbance reading at 405 nm on a microplate reader (Synergy HTX, BioTek Instruments).

### 2.11. Immunofluorescence

Raw264.7 cells were seeded onto coverslips in 24-well plates at a density of 1.0×10^5^ cells/well, pretreated with 6PP at 200 μM for 2 h, and then stimulated with LPS (0.5 µg/mL) for 15 min (or 8 h in the case of Nrf2 translocation experiments). Next, the cells were fixed using 4% paraformaldehyde (#PC2031-050-00, Biosesang), permeabilized with 0.1% Triton X-100 (#T8787, Sigma-Aldrich) for 10 min at RT, and then blocked using 1% BSA (in PBS) for 1 h at RT. The cells were then incubated with an anti-NF-κB/p65 or an anti-Nrf2 antibody at 4 °C overnight. The cells were then incubated with fluorescent secondary antibodies for 1 h in the dark. Finally, the coverslips were mounted onto glass slides using a mounting media (#S36936, Thermo Fisher Scientific Inc.). Then, we used a 1000× objective lens to acquire images and digitally zoomed to obtain images at a magnification of 3000× using software (NIS-Elements Advance Research V5.41.01, Nikon Instruments, Tokyo, Japan) under a confocal microscope (Nikon AX R, Nikon Instruments).

### 2.12. Statistical Analyses

Statistical analyses were done using SPSS Statistics 27 (IBM Corporation, Inc., New York, NY, USA). All data are presented as the mean ± standard error of the mean (SEM) of three independent experiments. Statistical significance was determined using the nonparametric Mann–Whitney U test. *p* < 0.05 indicates statistically significant differences.

## 3. Results

### 3.1. Isolation and Identification of 6PP

**Figure 1:**
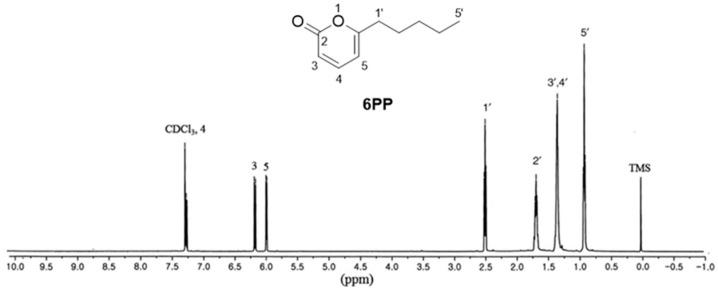
The chemical structure of 6-pentyl-α-pyrone (6PP) and its ^1^H-NMR spectrum.

Cultured *T. gamsii* was subjected to 100% MeOH extraction at RT. The MeOH extract was then successively partitioned using EtOAc, followed by silica gel column chromatography of the resulting EtOAc fraction to obtain sub-fractions. The sub-fractions were then subjected to LC–MS analysis using an in-house ultraviolet (UV) library database to identify one major compound. The sub-fraction containing the major compound was then separated on Sephadex LH-20, using C18 column chromatography, followed by isolation of the major compound using preparative HPLC. The isolated compound was identified as 6PP (Figure 1) by comparing its 1D NMR spectrum [^1^H-NMR (500 MHz, CDCl_3_) δ 7.26 (1H, dd, *J* = 6.5, 9.5 Hz), 6.15 (1H, d, *J* = 9.5 Hz), 5.97 (1H, d, *J* = 6.5 Hz), 2.48 (2H, t, *J* = 7.5 Hz), 1.64–1.70 (2H, m), 1.24–1.36 (4H, m), 0.90 (3H, t, *J* = 6.5 Hz) ppm] with previously reported data [13], as well as by analyzing the LC-MS data showing the molecular ion peak at *m*/*z* 167.1 [M + H]^+^ (Supplementary Figure S1). The structure was further confirmed through comparison with the standard available in LC-MS analysis.

### 3.2. Non-Cytotoxic 6PP Concentrations Modulate Intracellular GSH Levels

**Figure 2:**
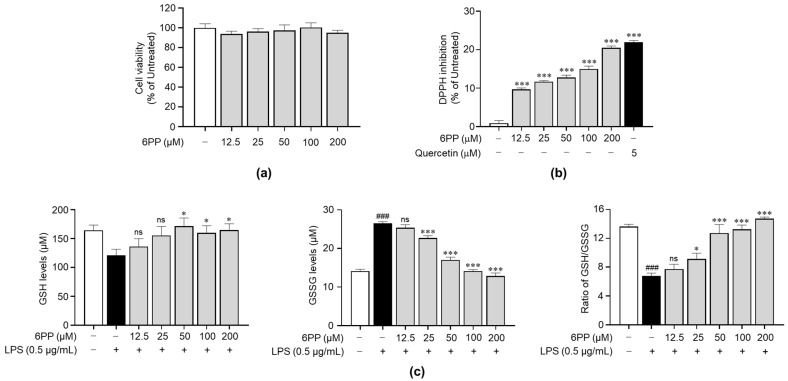
Reducing effects of 6PP under non-cytotoxic concentration. (**a**) Raw264.7 cells were treated with 6PP at indicated concentrations for 24 h, followed by cell viability analysis. (**b**) Inhibition of the free radical DPPH elicited by a range of 6PP concentrations (0–200 µM). Quercetin was used as the positive control. Statistical significance between the groups was determined using the Mann–Whitney U test. *** indicate *p* < 0.001 vs. untreated groups. (**c**) The cells were pretreated with 6PP at indicated concentrations for 2 h and then stimulated with LPS (0.5 µg/mL) for 24 h. Total GSH, GSSG, and the GSH/GSSG ratio were determined using a GSH assay kit. The data represent the mean ± standard error of the mean (SEM) of three independent experiments. Statistical significance between the groups was determined using the Mann–Whitney U test. ### indicates *p* < 0.001 in the untreated group vs. the LPS-treated group. * and *** indicate *p* < 0.05 and <0.001, respectively, in the LPS- vs. the 6PP-treated groups. ns indicates not significant. 6PP, 6-pentyl-α-pyrone; GSH, glutathione; GSSG, glutathione disulfide.

To identify its non-cytotoxic concentrations in Raw264.7 cells, we treated the cells with 6PP at 12.5, 25, 50, 100, and 200 μM for 24 h. This analysis revealed that when compared with the untreated group, 6PP was not significantly cytotoxic at the tested concentrations (Figure 2a). Therefore, subsequent experiments were conducted using 6PP at concentrations of up to 200 μM. Prior to identifying the antioxidant property of 6PP in cells, a DPPH assay was conducted to verify whether 6PP has direct antioxidant activity. As shown in Figure 2b, its radical scavenging activity was not as strong as quercetin, a well-known antioxidant molecule. Next, we investigated the potential antioxidant activity of 6PP in Raw264.7 cells following LPS-induced oxidative stress by assessing the intracellular levels of GSH and GSSG, as well as the GSH/GSSG ratio (Figure 2c). This analysis found that the LPS-treated group exhibited a reduction of GSH levels and GSH/GSSG ratio as well as the induction of GSSH. However, pretreatment with the indicated concentrations of 6PP showed notable increases in intracellular GSH levels and GSH/GSSG ratio. GSSG levels were also reduced by 6PP treatment in a dose-dependent manner.

### 3.3. 6PP Inhibits LPS-Induced Production of Inflammatory Mediators

**Figure 3:**
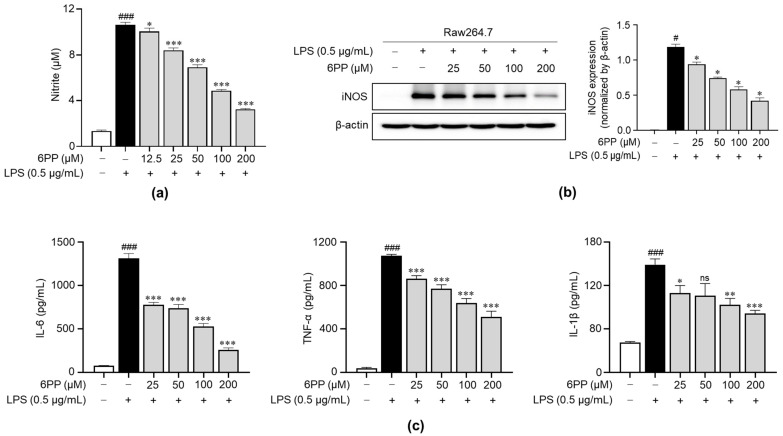
Inhibitory effects of 6PP on the production of proinflammatory mediators in LPS-stimulated Raw264.7 cells. Raw264.7 cells were pretreated at the indicated 6PP concentrations for 2 h and then stimulated with LPS (0.5 µg/mL) for 24 h. (**a**) The levels of NO in the cell culture media were quantified using Griess reagent. NO levels were determined by comparing its absorbance to that of a standard curve of nitrite standard solution concentrations. (**b**) iNOS protein levels were determined using western blotting, with β-actin as the loading control. (**c**) The levels of IL-6, TNF-α, and IL-1β were determined using ELISA. The data represent the mean ± SEM of three independent experiments. Differences between groups were compared using the Mann–Whitney U test. # and ### represent *p* < 0.05 and <0.001, respectively, vs. the LPS-untreated group. *, **, and *** indicate *p* < 0.05, <0.01, and <0.001, respectively, vs. the LPS-treated group. ns indicates not significant. NO, nitric oxide; iNOS, inducible nitric oxide synthase; IL, interleukin; TNF, tumor necrosis factor.

We examined the impact of 6PP on the production of NO upon stimulation with LPS. Data revealed that LPS triggered a significant increase in NO levels and that this effect was dose-dependently inhibited by pretreatment with 6PP (Figure 3a). Moreover, iNOS expression, a responsible enzyme for the production of NO [26,27,28], was reduced by 6PP treatment. To determine the anti-inflammatory potential of 6PP, we examined its ability to inhibit the secretion of proinflammatory cytokines, including IL-6, TNF-α, and IL-1β in Raw264.7 cells. LPS-induced production of proinflammatory cytokines was alleviated by pretreatment of 6PP (Figure 3c).

### 3.4. 6PP Exerts Anti-Inflammatory Effects by Activating HO-1

**Figure 4:**
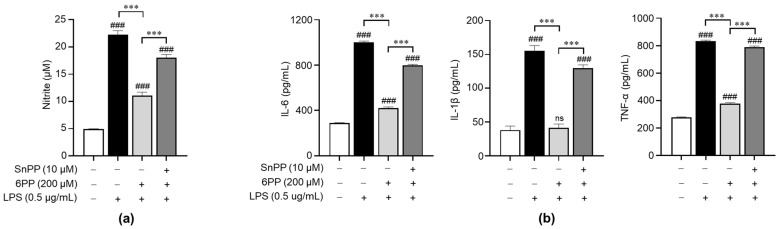
Involvement of antioxidant HO-1 in the 6PP-mediated regulation of proinflammatory mediators’ production. Raw264.7 cells were pretreated with 6PP (200 µM) for 2 h in the presence or absence of SnPP (10 µM), followed by LPS stimulation (0.5 µg/mL) for 24 h. (**a**) NO levels in the culture media were determined using the Griess assay and quantified against a standard curve of nitrite standard-solution concentrations. (**b**) The levels of IL-6, TNF-α, and IL-1β in the culture media were determined using ELISA. The data are presented as mean ± SEM of three independent experiments. Statistical differences between the groups were compared using the Mann-Whitney U test. ### indicates *p* < 0.001 vs. the LPS-untreated group. *** indicates *p* < 0.001 between paired groups. ns indicates not significant. HO-1, heme oxygenase-1; SnPP, Sn-protoporphyrin.

To investigate the relationship between the anti-inflammatory effects of 6PP and the activation of antioxidant signaling molecules, we treated LPS-stimulated macrophages with Sn-protoporphyrin (SnPP), a HO-1 inhibitor [29], and examined its effects on the production of NO and proinflammatory cytokines. SnPP significantly recovered 6PP-mediated inhibition of NO production (Figure 4a), indicating that HO-1 activity is involved in the regulation of 6PP-mediated NO production, at least in part. Furthermore, SnPP showed similar recovery effects for 6PP-mediated alleviation of proinflammatory cytokines’ production (Figure 4b).

### 3.5. 6PP Mitigates LPS-Induced Oxidative Stress by Activating the Nrf2 Signaling Pathway and Inducing Nrf2 Nuclear Translocation

**Figure 5:**
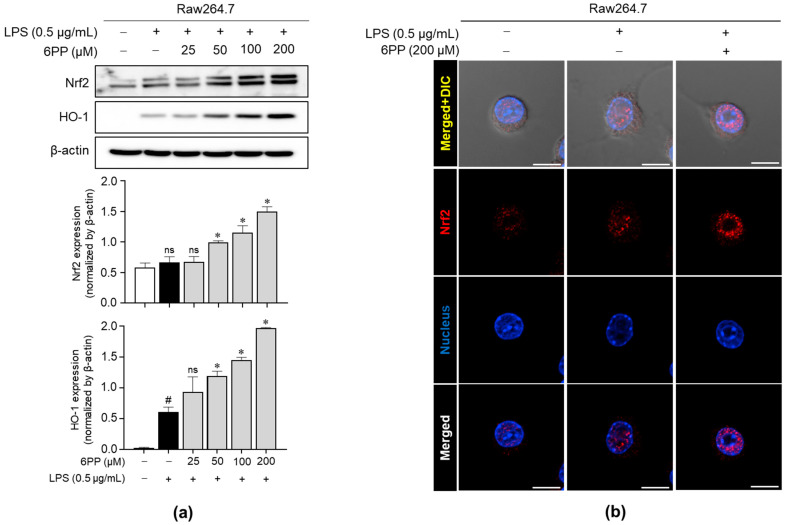
Enhancing effects of 6PP on the expression of Nrf2 and HO-1. (**a**) Raw264.7 cells were treated with LPS (0.5 µg/mL) in the presence of 6PP at the indicated concentrations for 24 h. iNOS protein levels were assessed using western blot analysis, with β-actin as the loading control. (**b**) Nrf2 (red) nuclear translocation was examined using confocal microscopy. Nuclei were counterstained with DAPI (blue). The data represent the mean ± SEM of three independent experiments. Statistical differences between the groups were compared using the Mann–Whitney U test. # indicates *p* < 0.05 vs. the LPS-untreated group. * indicates *p* < 0.05 vs. the LPS-treated group. ns indicates not significant. Scale bar: 10 µm. Nrf2, nuclear factor erythroid 2-related factor 2; DAPI, 4′,6-diamidino-2-phenylindole.

We investigated whether 6PP promotes the upregulation of critical antioxidant proteins, such as Nrf2 and HO-1, in Raw264.7 macrophages. As shown in Figure 5a, 6PP significantly induced the expression levels of Nrf2 and HO-1 in a dose-dependent manner. Moreover, immunofluorescence analysis revealed that 6PP significantly enhanced nuclear expression levels of Nrf2 (Figure 5b and Figure S2).

### 3.6. 6PP Inhibits IκB Phosphorylation and Degradation and NF-κB/p65 Nuclear Translocation

**Figure 6:**
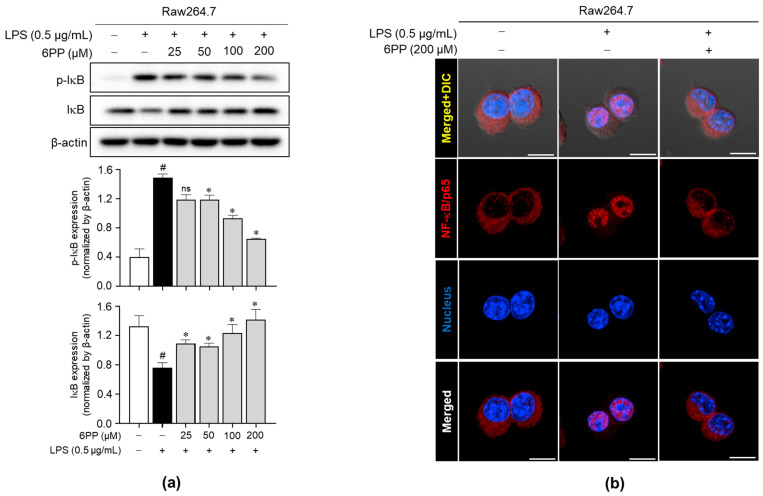
Inhibitory effects of 6PP on the NF-κB signaling pathway. Raw264.7 cells were pretreated with 6PP (200 µM) for 2 h and then stimulated with LPS (0.5 µg/mL) for 15 min. (**a**) Western blot analysis of the phosphorylated and total IκB levels, with β-actin as the loading control. (**b**) NF-κB/p65 (red) nuclear translocation was analyzed using confocal microscopy. Nuclei were counterstained with DAPI (blue). The data represent the mean ± SEM of three independent experiments. Statistical differences between groups were compared using the Mann–Whitney U test. # indicates *p* < 0.05 vs. the LPS-untreated group; * indicates *p* < 0.05 vs. the LPS-treated group. ns indicates not significant. Scale bar: 10 µm. IκB, an inhibitor of κB; NF-κB, nuclear factor kappa B.

To determine the involvement of the NF-κB signaling pathway in 6PP-mediated anti-inflammatory effects, the effect of 6PP on the IκB phosphorylation and degradation was investigated. Data revealed that 6PP reduces LPS-induced IκB phosphorylation and thereby blocks LPS-induced IκB degradation (Figure 6a). Moreover, LPS-induced NF-κB/p65 nuclear translocation was alleviated by 6PP treatment (Figure 6b and Figure S3).

### 3.7. 6PP Suppresses MAPK Phosphorylation

**Figure 7:**
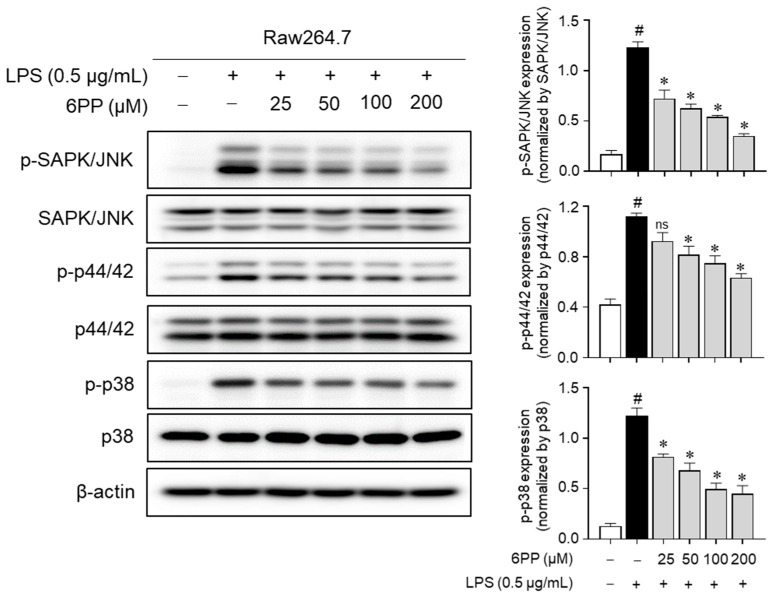
Inhibitory effects of 6PP on the MAPK phosphorylation. Raw264.7 cells were pretreated with 6PP at indicated concentrations for 2 h and then stimulated with LPS (0.5 µg/mL) for 15 min. The levels of phosphorylated and total MAPK signaling pathway factors (SAPK/JNK, p44/42, and p38) were assessed using western blotting, with β-actin as the loading control. The levels of p-SAPK/JNK, p-p44/42, and p-p38 were normalized to levels of SAPK/JNK, p44/42, and p38, respectively. The data represent the mean ± SEM of three independent experiments. Statistical differences between groups were compared using the Mann–Whitney U test. # indicates *p* < 0.05 vs. the LPS-untreated group. * indicates *p* < 0.05 vs. the LPS-treated group. ns indicates not significant. MAPKs, mitogen-activated protein kinase; SAPK/JNK, stress-activated protein kinase/c-Jun N-terminal kinases; p-, phosphorylated.

To determine whether 6PP-mediated inhibition of proinflammatory responses is regulated by MAPKs, changes in MAPKs’ phosphorylation level by 6PP were measured in LPS-treated Raw264.7 macrophages. Data showed that LPS-induced phosphorylation of MAPKs was alleviated by 6PP in a dose-dependent manner (Figure 7), implying that 6PP suppresses the production of proinflammatory mediators by inhibiting MAPKs’ activation as well as NF-κB in LPS-stimulated Raw264.7 cells.

## 4. Discussion

6PP is a secondary metabolite produced by fungi and an antifungal natural compound. This unsaturated lactone has a coconut-like smell and has applications as a food aroma enhancer. The pronounced antagonistic effects of 6PP against various fungi have been extensively studied [30,31]. Its ability to inhibit the growth of plant pathogenic fungi, protect from postharvest pathogens, and suppress mycotoxins has gained significant attention. Notably, 6PP has been shown to influence mycotoxins, such as deoxynivalenol and fusaric acid, which are produced by the *Fusarium* spp. [32].

In this study, we demonstrate that 6PP effectively suppresses LPS-induced inflammation and oxidative stress in Raw264.7 macrophages. Our analyses of the underlying molecular mechanisms indicate that 6PP inhibits the production of NO and proinflammatory cytokines by suppressing the activation of NF-κB and phosphorylation of MAPKs. Furthermore, we found that the Nrf2/HO-1 signaling pathway mediates the antioxidant activity of 6PP. To the best of our knowledge, this is the first study to comprehensively investigate the anti-inflammatory and antioxidant effects of 6PP, as well as their underlying mechanisms.

NO levels, which indicate inflammatory responses in LPS-stimulated macrophages, are primarily regulated by iNOS [33]. Our findings demonstrate that even at a low concentration (25 μM), 6PP potently inhibits NO production. Moreover, 6PP significantly suppressed iNOS protein levels in a concentration-dependent manner (Figure 3a,b). These findings suggest that 6PP attenuates NO production during LPS-induced inflammation by suppressing NOS. Inflammatory cytokines, which play a crucial role in systemic inflammation, are key inflammatory response mediators [34,35,36]. In this study, we found that 6PP suppressed LPS-induced production of the inflammatory cytokines IL-6, IL-1β, and TNF-α in a concentration-dependent manner (Figure 3c). Together, these findings indicate that 6PP effectively suppresses LPS-induced inflammatory responses.

Previous studies have shown a close association between proinflammatory mediators and NF-κB, a crucial regulator of inflammatory responses [37,38]. Upon stimulation by external factors, such as LPS, IκBα undergoes phosphorylation-dependent degradation [39], which triggers the nuclear translocation of activated NF-κB, which drives the expression of various proinflammatory mediators [40]. Thus, the modulation of NF-κB signaling has therapeutic potential against various inflammatory diseases. Our analyses of the mechanisms that underlie the anti-inflammatory effects of 6PP show that pretreating Raw264.7 macrophages with 6PP effectively suppresses LPS-induced IκB phosphorylation, thereby preventing the rapid degradation of IκB and inhibiting the nuclear translocation of the p65 subunit of NF-κB (Figure 6). Consequently, NF-κB signaling inhibition by 6PP suppresses the secretion of various proinflammatory mediators, resulting in anti-inflammatory effects. The MAPK signaling cascade also drives the expression of inflammatory mediators upon LPS stimulation. MAPKs, including JNK, ERK, and p38, are reported to upregulate proinflammatory cytokines, such as TNF-α, IL-6, IL-1β, and iNOS, in LPS-stimulated Raw264.7 macrophages [41]. Therefore, targeting this pathway has the potential to control LPS-induced inflammatory diseases. Our analysis of the impact of 6PP on the expression of MAPKs in LPS-stimulated Raw264.7 cells revealed that pretreatment with 6PP significantly suppresses the phosphorylation of JNK, p44/42, and p38. These findings suggest that 6PP exerts its anti-inflammatory effects by inhibiting MAPK phosphorylation.

Oxidative stress is characterized by a marked elevation of intracellular ROS levels [42]. Several studies have shown that oxidative stress is elevated in inflammatory diseases [43,44,45]. Antioxidant enzymes are closely associated with the transcription factor Nrf2, which plays a crucial role in the regulation of oxidative stress [46,47]. In this study, we observed that pretreatment with 6PP reduces GSH levels and the GSH/GSSG ratio (Figure 1). Since GSH is a representative intracellular antioxidant and is converted to GSSG when oxidative stresses occur, 6PP-induced increases in GSH level and GSH/GSSG ratio indicate that 6PP could be a candidate for the alleviation of excess oxidative stresses. Nrf2, a highly conserved transcription factor, is involved in the primary host defense system [48]. In unstimulated conditions, Nrf2 is sequestered in the cytoplasm by its negative regulator protein Keap1 [49]. However, upon extracellular stimulation, Nrf2 dissociates from keap1 and translocates into the nucleus, where it interacts with antioxidant response elements and induces the expression of cytoprotective proteins, such as HO-1, thereby counteracting oxidative stress [49,50,51]. HO-1, a member of the heat shock protein family, is a key antioxidant, anti-inflammatory, and cytoprotective enzyme, and its expression is regulated by Nrf2 [46]. To determine the antioxidant effects of 6PP, we investigated alterations in the Nrf2/HO-1 signaling pathway in LPS-stimulated Raw264.7 macrophages and found that 6PP strongly triggers Nrf2 nuclear translocation, thereby upregulating HO-1 expression and enhancing antioxidant enzyme activity (Figure 5). Furthermore, we found that SnPP, a HO-1 inhibitor, effectively reversed 6PP-mediated inhibition of LPS-induced production of NO and proinflammatory cytokines (Figure 4). These findings indicate that in Raw264.7 macrophages, 6PP activates the Nrf2/HO-1 signaling pathway, thereby mitigating LPS-induced oxidative stress response.

## 5. Conclusions

This study provides initial evidence that 6PP has anti-inflammatory and antioxidant effects in LPS-stimulated Raw264.7 macrophages. We demonstrate that 6PP modulates the NF-κB, MAPK, and Nrf2 signaling pathways, thereby suppressing the production of inflammatory mediators, such as NO and proinflammatory cytokines, as well as significantly increasing the levels of GSH and the GSH/GSSG ratio. Furthermore, 6PP triggers Nrf2 nuclear translocation, thereby inducing the expression of antioxidant enzymes. These results suggest that 6PP, a fungi-derived secondary metabolite, could be a beneficial candidate for protection against oxidative stresses and inflammatory states.

## Data Availability

Data are contained within the article and Supplementary Materials.

## Supplementary Materials

The following supporting information can be downloaded at: https://www.mdpi.com/article/10.3390/antiox12122028/s1, Figure S1: (A) UV chromatogram of LC/MS (detection wavelength was set as 254 nm) and (B) UV and MS data for 6-pentyl-α-pyrone (6PP); Figure S2: Effect of 6PP on the nucleus translocation of Nrf2; Figure S3: Effect of 6PP on the nucleus translocation of NF-κB.
