# Supplementary material for: 6-Pentyl-α-Pyrone from Trichoderma gamsii Exert Antioxidant and Anti-Inflammatory Properties in Lipopolysaccharide-Stimulated Mouse Macrophages
Source: Antioxidants (Basel). 2023 Nov 22;12(12):2028. doi: 10.3390/antiox12122028 (PMC10741142; doi:10.3390/antiox12122028)
Supplement: Supplementary file 1 [file antioxidants-12-02028-s001.zip › antioxidants-2698724-supplementary.pptx]

## Slide 1
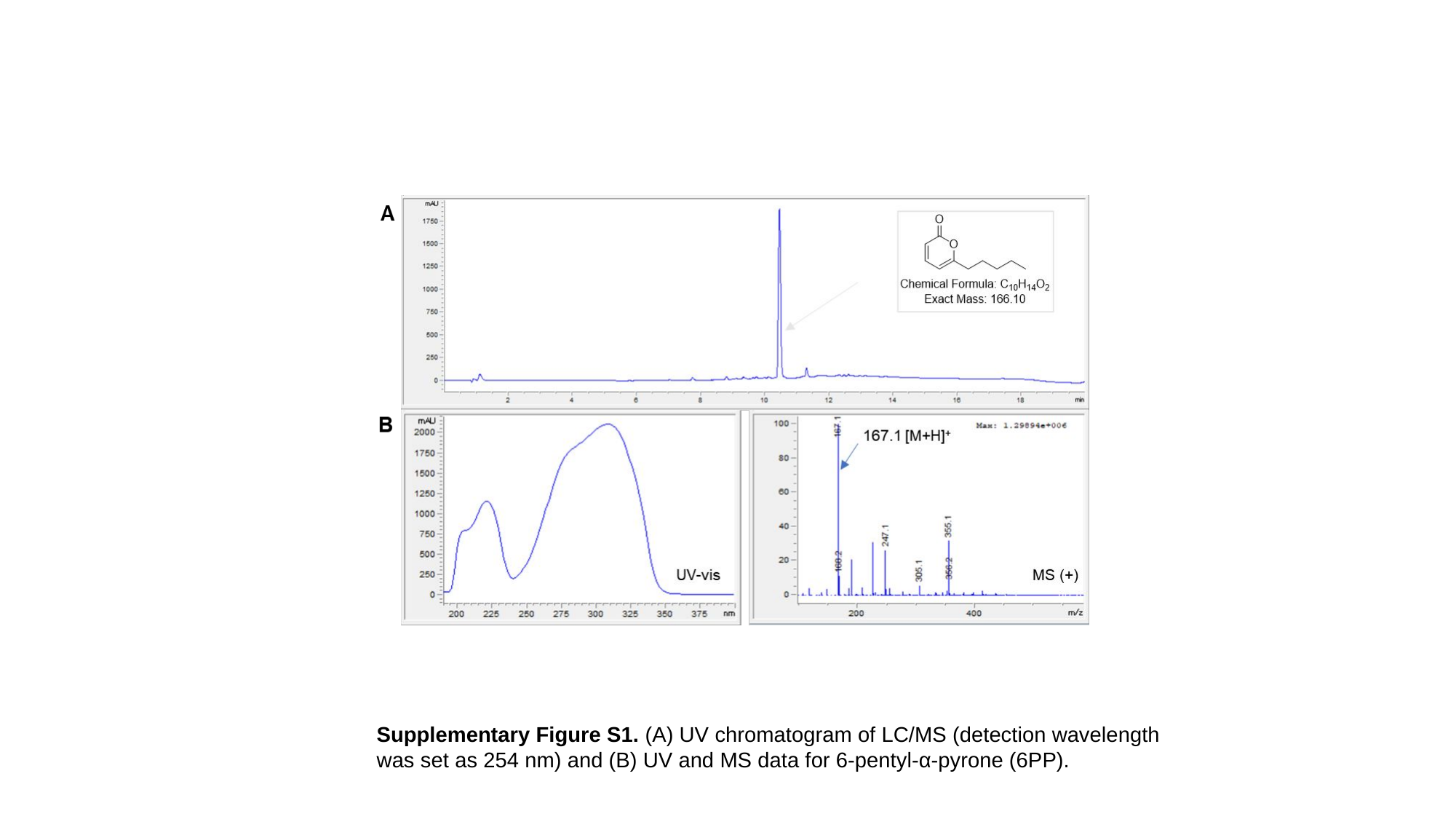

Supplementary Figure S1. (A) UV chromatogram of LC/MS (detection wavelength was set as 254 nm) and (B) UV and MS data for 6-pentyl-α-pyrone (6PP).

## Slide 2
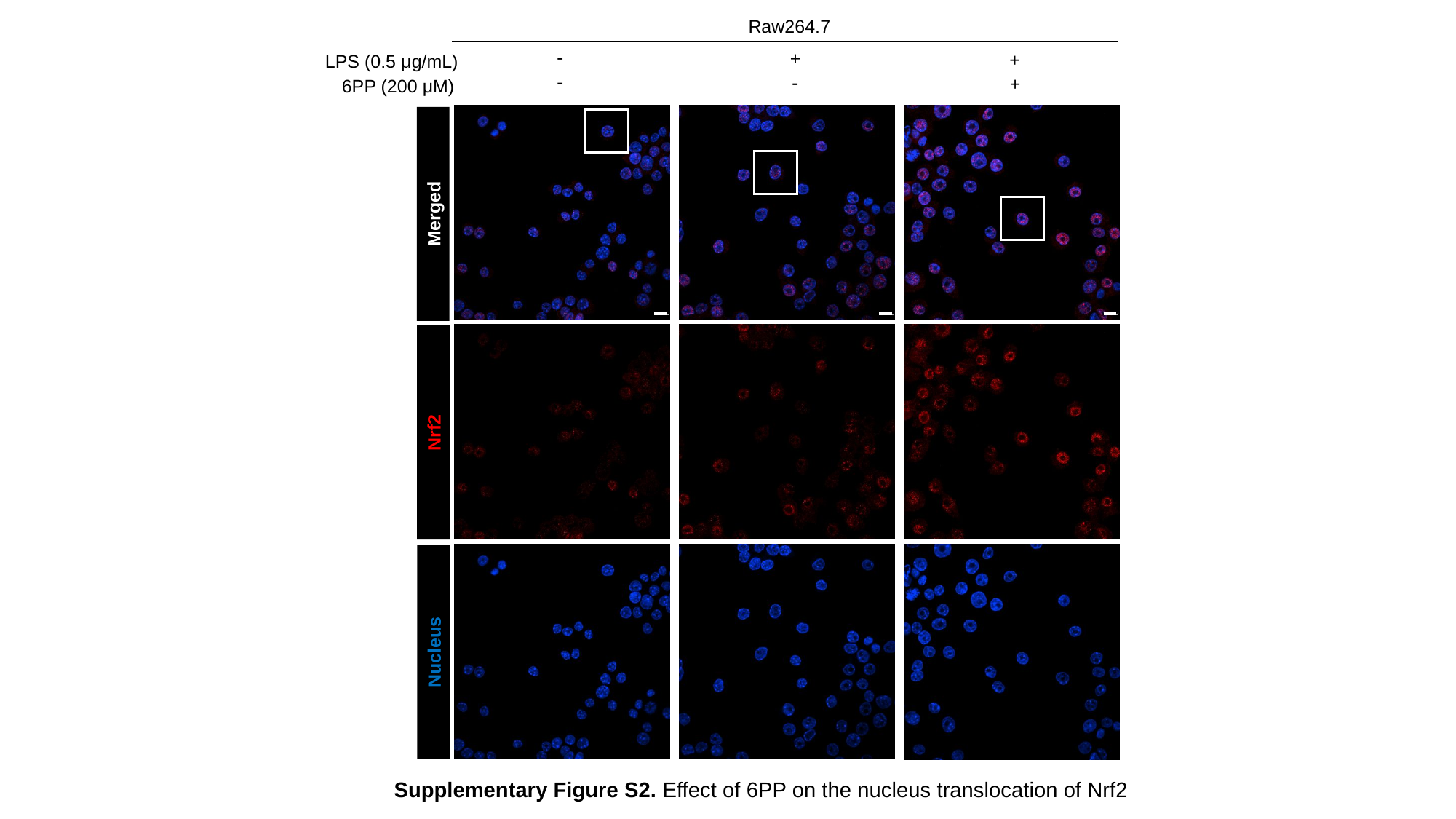

Raw264.7
−
+
+
LPS (0.5 μg/mL)
−
−
+
 6PP (200 μM)
Merged
+
Nrf2
Nucleus
Supplementary Figure S2. Effect of 6PP on the nucleus translocation of Nrf2

## Slide 3
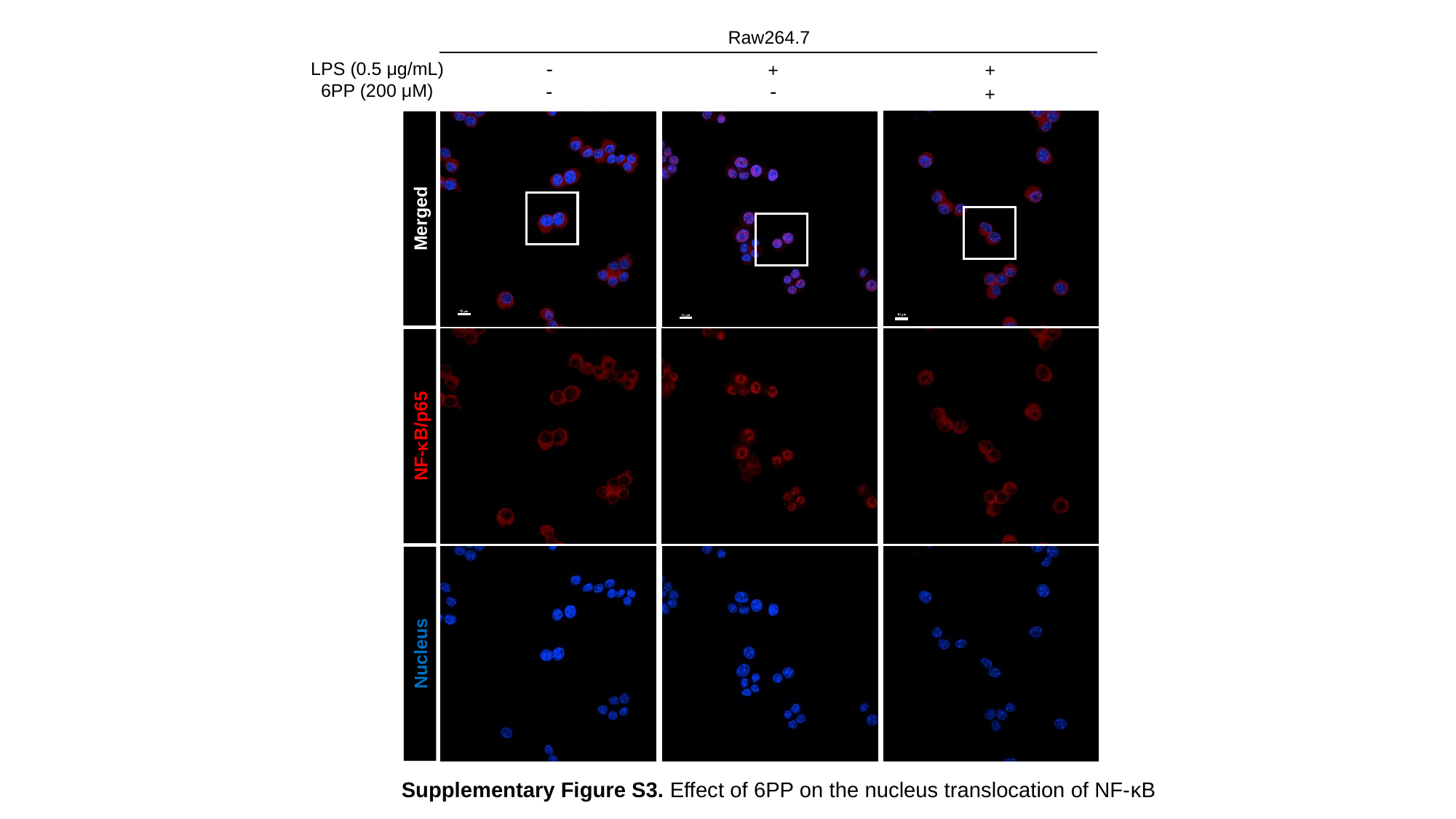

Raw264.7
LPS (0.5 μg/mL)
−
+
+
 6PP (200 μM)
−
−
+
Merged
NF-κB/p65
Nucleus
Supplementary Figure S3. Effect of 6PP on the nucleus translocation of NF-κB
